# PM_2.5_ Concentration Prediction: Ultrahigh Spatiotemporal Resolution Achieved by Combining Machine Learning and Low-Cost Sensors

**DOI:** 10.3390/s25175527

**Published:** 2025-09-05

**Authors:** Junfeng Li, Jiaqi Chen, Ran You, Qingqing He

**Affiliations:** School of Resources and Environmental Engineering, Wuhan University of Technology, Wuhan 430070, China; 343631@whut.edu.cn (J.L.); 343613@whut.edu.cn (J.C.); 343683@whut.edu.cn (R.Y.)

**Keywords:** low-cost sensors, machine learning, ultrahigh-spatiotemporal-resolution prediction, spatiotemporal analysis

## Abstract

PM_2.5_ pollution is still serious in densely populated cities with frequent traffic activities, and it continues to threaten public health. Therefore, it is urgent that we obtain ultrahigh-resolution data that can reveal its complex spatiotemporal variation characteristics, supporting more refined environmental governance and health risk prevention and control. This study first carried out ground monitoring based on low-cost sensors combined with observation results, which were corrected with the national environmental monitoring station data. This study also introduced multi-source auxiliary variables and constructed a machine learning model through the stacking ensemble learning method. The model combines corrected low-cost sensor data with high-resolution prediction factors to achieve ultrahigh-spatiotemporal-resolution prediction of PM_2.5_ at 100 m × 100 m spatial resolution and hourly temporal resolution. The results show that the constructed model shows good prediction ability in 5-fold cross validation, with an overall R^2^ of 0.93 and a root mean square error (RMSE) of 3.09 μg/m^3^. The spatiotemporal analysis based on the prediction results further revealed that the PM_2.5_ concentration in the city showed significant variation characteristics at both the ultra-local scale and the short-term scale, reflecting the high heterogeneity of urban air pollution. In addition, by comparing and analyzing the monitoring data of a national environmental monitoring station that were not used in the correction, it was found that the corrected low-cost sensor data significantly reduced the prediction uncertainty, reducing the RMSE from 72.068 μg/m^3^ to 16.759 μg/m^3^, verifying its effectiveness in high spatiotemporal resolution air quality monitoring. This shows that low-cost sensors are expected to make up for the problem of insufficient spatial coverage in traditional national environmental monitoring stations, supporting the successful assessment of urban-level air pollution and health risk management, and therefore having broad application prospects.

## 1. Introduction

Air pollution is widely recognized as a significant public health risk. According to statistics, about 4.2 million people die prematurely each year due to exposure to air pollution worldwide, particularly fine particulate matter (PM_2.5_), which is closely related to the high incidence of respiratory and cardiopulmonary diseases [1]. Studies have shown that, even if PM_2.5_ concentrations are lower than the national standard limit, they may still pose significant health risks [2,3,4,5]. In China, air pollution control is particularly arduous due to the complex types and dispersed spatial distribution of pollution sources [6]. In recent years, after implementing continuous pollution-prevention and -control measures, China’s overall air quality has improved. However, due to the density of China’s populations, frequent traffic activities, and diverse distribution of pollution sources, PM_2.5_ pollution levels in urban areas are still high.

The complex underlying surface characteristics within cities further exacerbate the spatiotemporal heterogeneity of PM_2.5_, causing its concentration to fluctuate dramatically in short-term and local spatial scales. Brokamp et al. [3] pointed out that compared with regional concentration differences, the impact of microscale PM_2.5_ concentration changes on health may be more significant. Therefore, accurately capturing the short-term and local changes in PM_2.5_ in cities is of great significance for assessing exposure risks and formulating precise prevention and control strategies. This requires a concentration dataset with ultrahigh spatiotemporal resolution to reflect the pollution characteristics in the urban microenvironment.

At present, China’s air quality monitoring system mainly relies on the sparsely distributed national environmental monitoring stations in the National Environmental Monitoring Centre (CNEMC). Although national environmental monitoring stations can provide high-temporal-resolution data at the hourly level, the number of stations is limited and sparsely distributed in urban areas due to the high cost of station construction and maintenance [7,8,9]. Therefore, it is difficult to effectively reveal the fine spatial distribution of PM_2.5_ concentrations in cities, especially in suburban and urban fringe areas. Considering that great difficulties are encountered in setting up more monitoring points in terms of cost and practical operation, it is urgent that we use statistical modeling methods to predict and generate PM_2.5_ concentration datasets with ultrahigh spatiotemporal resolution based on existing monitoring data and auxiliary information, thereby providing support for urban air pollution prevention and control and health risk assessment.

In the above context, this study combines machine learning methods with high-spatiotemporal-density monitoring data from low-cost sensors to develop a prediction model for PM_2.5_ concentrations with ultrahigh spatiotemporal resolution based on 100 m × 100 m spatial resolution and hourly temporal resolution. Additionally, this study explores the spatiotemporal distribution variation characteristics of PM_2.5_ concentrations within cities at a local scale. This study makes up for the following limitation: the existing air pollution monitoring system cannot capture local changes in PM_2.5_ concentrations due to insufficient spatial resolution. Accordingly, the findings presented here enrich the methods and systems for the predictive modeling of air pollutant concentration monitoring with ultrahigh spatiotemporal resolution at a lower cost, promoting the development of refined exposure assessments and health risk research on air pollution.

## 2. Literature Review

In order to achieve the prediction of the spatiotemporal distribution of air pollutant concentrations at an urban scale, many scholars have proposed a variety of modeling methods, including pollutant–meteorological stepwise regression models, models designed for the numerical simulation of atmospheric chemistry, diffusion models, and land use regression (LUR) models. Among them, statistical modeling methods based on site monitoring data, especially regression-algorithm-driven models, have become one of the most widely used prediction methods because of their relatively simple implementation, moderate data requirements, and ability to effectively capture spatial heterogeneity [1,6,10]. Compared with other methods, such as the numerical simulation of atmospheric chemistry, statistical modeling has significant advantages in spatial resolution and model flexibility. It is particularly suitable for urban areas with limited sample points. It can estimate pollutant concentrations at a smaller scale and make up for a lack of monitoring network coverage [11]. This type of method usually relies on three core elements: (1) monitoring data of the target variable, (2) high-resolution prediction variables that reflect the spatiotemporal changes in pollution, and (3) regression algorithms used in modeling. This section will systematically review the research progress of PM_2.5_ concentration prediction based on statistical modeling from the above three aspects, and provide theoretical support for subsequent model development and method selection.

### 2.1. Target Variable

As the target variable in statistical modeling, the spatial distribution and quality of air pollutant concentration monitoring data directly affect the prediction accuracy of the model. To build a prediction model with high spatial resolution, it is necessary to rely on high spatial density monitoring data support. Otherwise, although the model output has high resolution in form, it may not be able to accurately reflect the local pollution characteristics in fact, and may affect the accuracy of the prediction modeling [12,13].

Although China’s existing national environmental monitoring stations can provide continuous and high-quality monitoring data, due to the high cost of construction and operation and maintenance, their spatial layout is relatively sparse, meaning that it is difficult to meet the spatial resolution requirements of refined exposure assessment within cities [14]. To address this limitation, low-cost sensors have attracted attention as a supplementary means in recent years. This type of sensor has the advantages of flexible deployment, low cost, and wide coverage. It can be used to obtain high-spatial-density pollutant concentration observation data and provide support for ultrahigh-resolution modeling. However, low-cost sensors have certain differences in measurement accuracy and stability, and are easily affected by environmental factors. Therefore, it is necessary to use reasonable correction methods to improve data quality and ensure their effectiveness and reliability when used as target variables [12].

### 2.2. Prediction Variables

In statistical modeling, prediction variables are used to describe the generation, diffusion, and accumulation of pollutants. Their spatiotemporal distribution characteristics significantly affect the model’s ability to describe changes in pollutant concentrations and its prediction accuracy. Prediction variables that affect changes in PM_2.5_ concentrations mainly include two categories: emission-related factors and meteorological driving factors. Common emission-related variables are represented by land use types, which are often constructed through GIS-derived data. They reflect human activities and natural environmental characteristics such as industrial land, transportation network, and vegetation cover, and are key indicators for characterizing differences in the spatial distribution of PM_2.5_ [15]. Such variables have high spatial resolution and are suitable for use in capturing microscale spatial heterogeneity within cities. However, their temporal update frequency is low, making it difficult for them to dynamically reflect short-term fluctuations in pollutant concentrations. To fill the information gap in the time dimension, meteorological variables are often introduced into the model to describe the key conditions that affect the generation, reaction, and diffusion of PM_2.5_, including wind speed, temperature, relative humidity, and planetary boundary layer height [16,17]. Such variables usually have a high temporal resolution (e.g., hourly), but their spatial resolution is relatively low, making it difficult to accurately depict fine-scale meteorological changes within cities. In addition, remote sensing data are also widely used in modeling, especially products such as aerosol optical depth (AOD), which can provide information on the spatial and temporal distribution of pollutants to a certain extent [18]. Taking MODIS as an example, its AOD data have a daily update frequency and a kilometer-level spatial resolution [19], but it is still difficult for them to take into account both high spatial and high temporal resolution, and there are limitations such as insufficient spatial granularity or observation discontinuity.

In summary, different types of prediction variables have their own advantages and disadvantages in terms of temporal and spatial resolution. Currently, there is no single data source that can simultaneously meet the needs of ultrahigh-spatiotemporal-resolution prediction of PM_2.5_ concentration. Therefore, in the actual modeling process, it is necessary to comprehensively utilize multi-source heterogeneous data and integrate their complementary information to enhance the model’s ability to characterize complex spatiotemporal variation characteristics.

### 2.3. Model Development and Construction

In the spatiotemporal prediction modeling of air pollutant concentrations, the choice of modeling method directly determines the model’s ability to express complex pollution-generation mechanisms and their predictive performances. Early studies mostly used linear regression models, such as multivariate linear regression (MLR) or geographically weighted regression (GWR), which have the advantages of simple form, high computational efficiency, and easy result interpretation [4,6,11]. However, these methods usually assume that there is a linear relationship between pollutants and predictors, which makes it difficult to capture the nonlinear mechanisms that are prevalent in reality, and their prediction accuracy and generalization ability are limited. In recent years, the generalized additive model (GAM) has been gradually introduced into air quality modeling in response to the nonlinear relationship between pollutant concentrations and driving variables [4,6]. This method can improve fitting accuracy while retaining the model’s interpretability by fitting nonlinear smooth functions to the variables, allowing researchers to identify the marginal effects and potential mechanisms of the variables. However, GAM still has certain limitations in variable interaction modeling and high-dimensional feature selection.

With the growth in data volume and the development of algorithms, machine learning methods have become one of the mainstream means of pollutant concentration prediction due to their excellent nonlinear fitting ability and high-dimensional feature processing ability [3,6,20]. Models such as random forest (RF), gradient boosting (such as XGBoost), and deep neural networks can automatically capture the complex nonlinear relationship between variables and significantly improve their prediction accuracy [21,22]. For example, Jain et al. [15] integrated RF with the LUR model to improve the precision of spatial distribution prediction; Yang et al. [23] constructed a multi-output random forest (MORF) model to estimate six air pollutants, with a prediction R^2^ of 0.94. Current research trends are gradually focusing on combining machine learning algorithms with multi-source high-spatiotemporal-resolution data to build more sophisticated and robust pollutant prediction models. Among them, XGBoost, as an efficient integrated learning method, has outstanding performance in air quality modeling due to its advantages in feature selection, nonlinear modeling, and training efficiency [20], providing strong technical support for the realization of ultrahigh-spatiotemporal-resolution PM_2.5_ prediction.

Overall, the current modeling research on ultrahigh-spatiotemporal-resolution PM_2.5_ concentration data at the urban scale is still relatively limited. Based on the inspiration of the target variables, predictor variables and modeling methods in the above review, this article will comprehensively apply the high-spatiotemporal-density PM_2.5_ monitoring data obtained by low-cost sensors, multi-source high-resolution predictor variables, and machine learning algorithms with nonlinear modeling capabilities to develop an ultrahigh-spatiotemporal-resolution PM_2.5_ concentration prediction model that is suitable for urban-scale applications. The goal is to achieve the continuous prediction of PM_2.5_ concentration with a spatial resolution of 100 m × 100 m and an hourly time resolution within the study area and monitoring period, providing support for urban pollution exposure assessment and refined management.

## 3. Materials and Methods

### 3.1. Study Area

**Figure 1:**
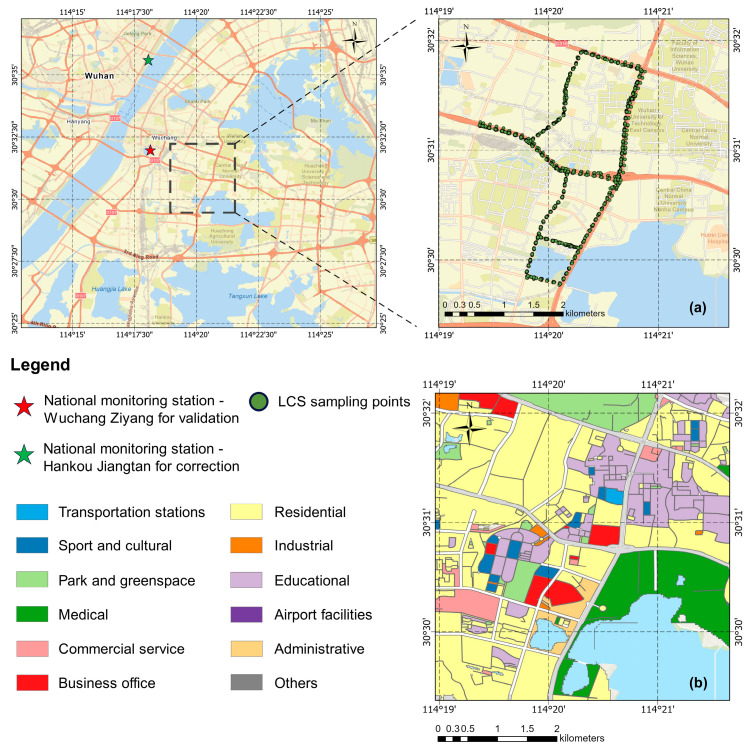
Study area map. (**a**) PM_2.5_ sampling points; (**b**) zoning of the territory.

The study area was an area in Hongshan District, Wuhan City, Hubei Province, China. This area contains typical commercial areas, residential areas, and educational areas, as shown in Figure 1b, and is passed by multiple main roads. There are some potential pollution sources, such as bus stations and storage centers.

The route covered by the green sampling points in Figure 1a is the designed monitoring route. The total length of the route is about 12 km, and the monitoring vehicles are bicycles and electric vehicles. The route covers main roads, secondary roads, and trunk roads, with main roads and secondary roads being the main ones (the trunk roads and main roads basically overlap on this route). The nearby areas include commercial areas (such as WS Dream Plaza in the north), residential areas (the areas near the route are mostly residential areas), school areas (primary schools, middle schools, and universities), and possible pollution sources (such as the passenger bus station in the west and Zhongbai Warehouse in the south, because of the possible entry and exit of large oil trucks). The spatiotemporal changes in the concentrations of air pollutants on different typical underlying surfaces can be studied at the same time.

The national environmental monitoring stations include the Hankou Jiangtan National Environmental Monitoring Station and the Wuchang Ziyang National Environmental Monitoring Station. The Hankou Jiangtan National Environmental Monitoring Station is used to correct low-cost sensors data, while the Wuchang Ziyang National Environmental Monitoring Station is used to verify the prediction results.

**Figure 2:**
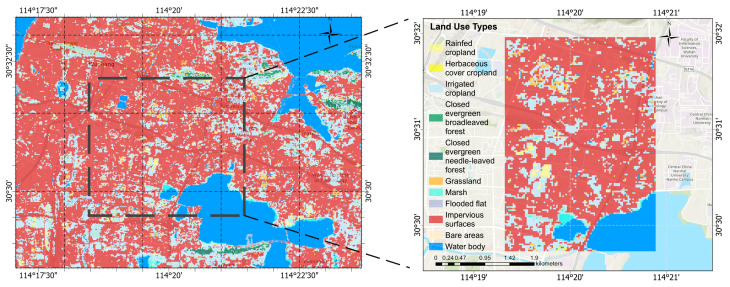
Land cover type distribution map of the study area.

The land cover in the study area is shown in Figure 2.

### 3.2. LCS Monitoring Campaign

#### 3.2.1. Monitoring Time Period

We conducted sampling monitoring activities along the monitoring route from 6:00 to 23:00 on 31 October, 1 November, 2 November, and 4 November 2024. Table 1 shows our monitoring schedule, where the time period corresponding to the “√” symbol indicates the monitoring period.

#### 3.2.2. Monitoring Tools

(1) Low-Cost Sensors

**Figure 3:**
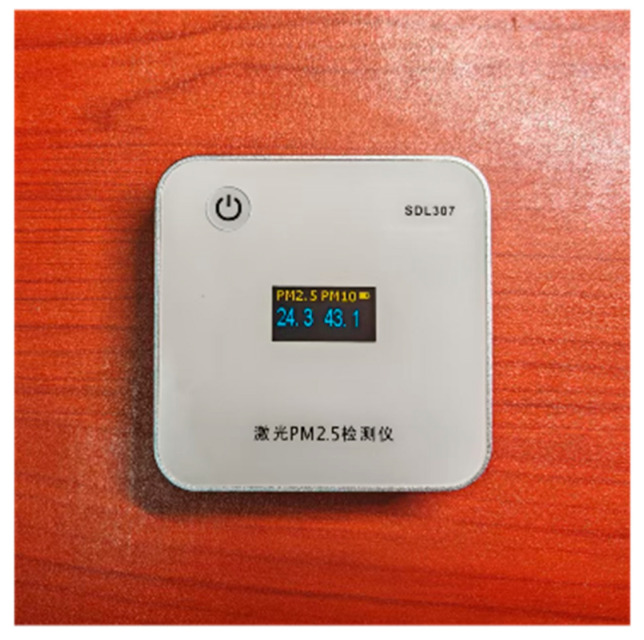
The low-cost sensor of model SDL307.

We purchased four SDL307 laser PM_2.5_ detectors produced by Nova Fitness Co., Ltd. in Jinan, Shandong, China, and used them as convenient low-cost sensors to collect PM_2.5_ concentration data in this study. The low-cost sensor detects data through laser detection method and can monitor PM_2.5_ and PM_10_ at the same time, with a resolution of 0.1 μg/m^3^, a maximum relative deviation of ±20%, and a response speed of seconds, recording data every 1 s. The low-cost sensor is shown in Figure 3.

This low-cost sensor was selected through a preliminary experiment in the study by Xu et al. [24], where the authors participated in a monitoring campaign similar to ours; its comparison with the reference-level instruments at the national environmental monitoring stations achieved very good results. In our study, the correlation analysis between the four purchased low-cost sensors, the reference-level instruments at the national environmental monitoring station (all above 0.90), and the correction results of these low-cost sensors in Section 4.1 also proved that this low-cost sensor could achieve accurate monitoring.

(2) Temperature and humidity data loggers

**Figure 4:**
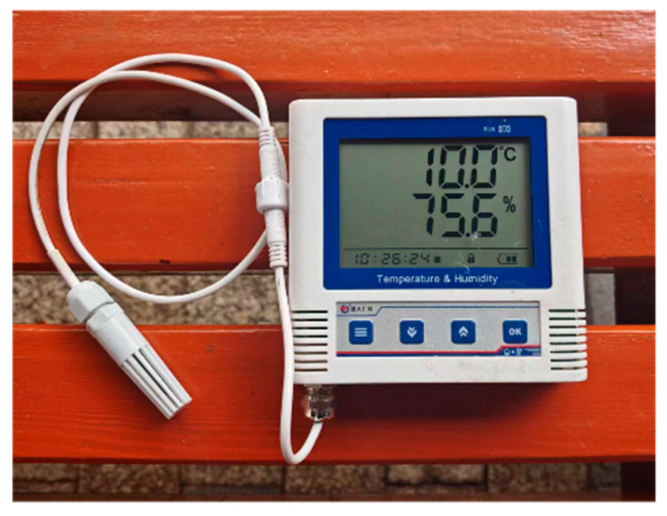
The temperature and humidity logger.

We also purchased four temperature and humidity loggers produced by Shandong Renke Control Technology Co., Ltd. in Jinan, Shandong, China. The monitoring range of temperature and relative humidity of the logger is −40~+80 °C and 0~100%, respectively, and the accuracy of the temperature and relative humidity measurements reaches ±0.1 °C and ±1.5%, respectively. The shortest interval setting allows data to be recorded every 5 s. The temperature and humidity logger is shown in Figure 4.

(3) GPS positioning tool

In order to obtain GPS positioning data during the monitoring period, we used the line-tracking function of the mobile phone software GPS Toolbox. When the satellite signal is well received, the positioning error of the software does not exceed 4~6 m.

For the entire monitoring process, before the monitoring is conducted, we recruited four monitoring personnel every hour to collect high-spatial-density data with different low-cost sensors (sampling interval is about 100 m). It is important to test the activities of the low-cost sensors before conducting the monitoring process; here, we found that the monitoring concentration of this low-cost sensor model in the state of low-speed movement (below 25 km/h) would be abnormally high. In the process of slowing down or stopping monitoring, the concentration would slowly decrease until it stabilized. In other words, monitoring concentration would only be normal when the sensors are relatively still. In the process from slowing down from a lower speed to a stop, and then to reach a stable monitoring concentration, takes between a few seconds and thirty seconds. Therefore, we stipulated that monitoring personnel must stop and conduct monitoring for 30 s every time they arrive at a sampling point, before they go on to the next sampling point. Bicycles or electric scooters were used as the means of transportation for this monitoring campaign; accordingly, the sampling height relative to the ground was approximately 1.15 m, which is lower than the average breathing height of pedestrians on the roadside. At each sampling point, in addition to monitoring PM_2.5_ concentrations with low-cost sensors, temperature and relative humidity data were recorded using temperature and humidity data loggers, and the geographic coordinates of each actual sampling location were recorded using GPS Toolbox software. After testing during the monitoring process, the monitored sections could basically receive GPS signals normally. In all monitoring activities, we collected a total of 2023 sampling data points, including 25,291 s of data.

### 3.3. Correction of Low-Cost Sensor Data

#### 3.3.1. Data Used for Correction

There are certain differences in monitoring quality between low-cost sensors and existing air quality reference-level monitoring instruments (e.g., monitoring instruments at national environmental monitoring stations have high accuracy). There are also small differences in the quality of the data collected by different individuals. Therefore, in order to ensure that the data monitored using low-cost sensors have the same reference standard, low-cost sensor monitoring data should be corrected based on the data of the monitoring instruments at national environmental monitoring stations.

**Figure 5:**
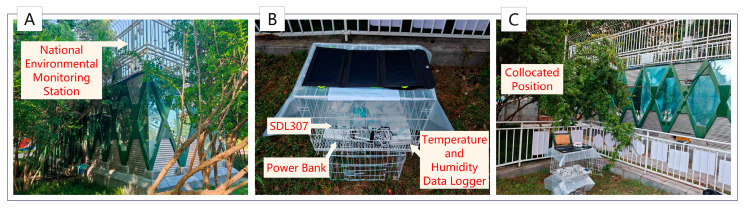
Co-location of low-cost sensors, temperature and humidity data loggers, and national environmental monitoring station instruments. (**A**) National Environmental Monitoring Station; (**B**) an assembly used to place the low-cost sensors and the temperature and humidity data loggers for collecting correction data. The low-cost sensors were powered by portable power banks and placed together with the temperature and humidity data loggers inside a large wire mesh ventilated cabinet; (**C**) co-location of the assembly for collecting correction data with the reference instruments at the National Environmental Monitoring Station.

We co-located the data collected using the low-cost sensors and the temperature and humidity data loggers with those collected using reference-level monitoring instruments at the National Environmental Monitoring Station in Hankou Jiangtan, Wuhan City. This was achieved during multiple time periods on days without heavy rainfall or haze, and without interference from external human activities. The time period for the co-cocated data was from 10 September 2024, to 30 October 2024. That is, the low-cost sensors, the temperature and humidity data loggers, and the national environmental monitoring station instruments were used to collect data through monitoring the same locations simultaneously. The low-cost sensors and temperature and humidity data loggers were placed about 1.5 m away from the national environmental monitoring station in a location with little human interference. They were placed approximately 0.6 m above the ground, with 360-degree horizontal ventilation around all the sampling ports. The national environmental monitoring station instruments were located approximately 3.6 m above the ground. Due to limited conditions, the low-cost sensors and temperature and humidity data loggers were not placed at the same height as the national environmental monitoring station instruments. The co-location diagram is shown in Figure 5.

Taking the instruments of the national environmental monitoring station as the reference instruments, the data collected by the low-cost sensors and temperature and humidity data loggers were fused with the data collected by the national environmental monitoring station instruments. Accordingly, we were able to construct correction models for the monitoring data collected by each low-cost sensor, and these models were used to correct the monitoring data of their corresponding low-cost sensors. The total numbers of effective hours of data collected by each low-cost sensor for correction are shown in Table 2.

The monitoring data collected using the national environmental monitoring station instruments during the co-location period were collected on the China National Environmental Monitoring Center (CNEMC) (https://air.cnemc.cn:18007/) and China Air Quality Historical Data (https://quotsoft.net/air/) platforms.

#### 3.3.2. Correction Method for Low-Cost Sensor Data

The data collected during the co-location period, including the monitoring data of the instruments at the national environmental monitoring station and the monitoring data of each low-cost sensor and temperature and humidity data logger, were integrated to develop an individual multiple linear regression correction model for each low-cost sensor. In these models, the data from the national environmental monitoring station instruments served as the target variables, and the data from the low-cost sensors and temperature and humidity data loggers served as predictors. As there were four low-cost sensors and all of them were equipped with temperature and humidity data loggers, a total of four correction models were developed.

The multivariate linear regression model is a statistical model used to establish the relationship between multiple independent variables and dependent variables. This model is based on a linear hypothesis, assuming that there is a linear relationship between the independent variables and the dependent variables, and the least squares method is used to estimate the parameters of the model. The multivariate linear regression model can capture the comprehensive impact of multiple independent variables on the dependent variable. Given the independent variables, the dependent variable can be predicted, and the impact of the independent variables on the dependent variable can be evaluated based on the parameters corresponding to the independent variables. The general form of the multivariate linear regression model is as follows:

(1) $$Y=\beta_{0}+\beta_{1}X_{1}+\beta_{2}X_{2}+\ldots +\beta_{n}X_{n}+\varepsilon_{n}$$

The correction results of the data for each low-cost sensor are presented in Section 4.1.

### 3.4. Prediction Variables

#### 3.4.1. Land Cover Data

Here, we used the 2022 global 30-m-resolution land cover dynamic product (GLC_FCS30D), released by Liu et al., from the Aerospace Information Research Institute, Chinese Academy of Sciences (https://doi.org/10.5281/zenodo.15063683). The description of the land use data is shown in Table 3.

#### 3.4.2. Road Network Data

Since traffic emission sources are important sources of PM_2.5_ emissions, we also collected traffic network data from the Open Street Map (https://www.openstreetmap.org/) platform. Open Street Map, referred to as OSM, is an open-source map data community, from which we obtained road network data for 2024.

#### 3.4.3. Meteorological Data

From the Environmental Meteorological Service Platform (http://eia-data.com/pm_history-html/), we obtained five types of meteorological data, including hourly temperature, wind speed, wind direction, relative humidity, and atmospheric pressure, from all meteorological stations in Wuhan and its surrounding 16 cities during the entire monitoring period, totaling data from 81 meteorological stations. The data are derived from the three-hourly data of China’s ground basic meteorological observation data from the China Meteorological Data Network, combined with the ECMWF reanalysis data, and interpolated into hourly meteorological station data through machine learning algorithms. In addition, data for the planetary boundary layer height reanalysis, with a resolution of 0.25° per hour from ERA5 during the study period, were collected.

The statistical results of all prediction variables are shown in Table 4.

### 3.5. Modeling Data Preprocessing Method

#### 3.5.1. Preprocessing of Sampling Point LCS Data

Before conducting monitoring activities, we corrected the recording time of the temperature and relative humidity data and the GPS positioning data. Then, we matched the monitoring data of each sampling point. Since the GPS positioning data had random fluctuations within the error, even if it was kept at a fixed sampling point for continuous monitoring, the positioning data were not always consistent. Therefore, we set a scheme to determine the sampling point and its monitoring data. Specifically, we found all the positioning points within 12 m of the monitoring route with a time difference greater than 12 s but not more than 100 s; here, we used the average longitude and latitude coordinates of these positioning points as the coordinates of the sampling points. The start and end times obtained from all the positioning points were used as the monitoring time of the sampling points. Since the recorded positioning times were in UTC, the corresponding hour plus 8 h was converted into Beijing time. We also added the requirement that, if two or more sampling points appeared in the same sampling within 12 m and the spatial distance between the sampling points was less than 8.5 m, then these sampling points were merged; accordingly, the final sampling point would be their average coordinates, and the monitoring time period was their union. Low-cost sensors have a characteristic in which the concentration slowly decreases until it stabilizes during the process of slowing down or stopping; therefore, we took the average data value of a maximum continuous period as the PM_2.5_ monitoring value of the sampling point in that hour. This data value contains the minimum value and the standard deviation of the monitoring data point (not exceeding 1 μg/m^3^ in the monitoring time period of each sampling point). Since the temperature and humidity data logger recorded the temperature and humidity data every 5 s, we filled in the missing seconds of temperature and humidity data with the temperature and humidity data of the nearest time point before it, and averaged the temperature and humidity data over this time period. Finally, the low-cost sensor monitoring data and temperature and relative humidity data in the monitoring time periods of all the sampling points were matched according to time; then, the PM_2.5_ monitoring data of each sampling point were corrected using the established correction model.

Then, the data were cleaned to remove the data corresponding to the abnormal values and the repeated time in the monitoring time period of all the sampling points; the time periods corresponding to the missing values were also removed.

#### 3.5.2. Meteorological Data Preprocessing

Using the inverse distance weighting method, we spatially interpolated the values of the five meteorological attributes of all meteorological stations every hour and resampled the planetary boundary layer height data. We previously established a geotif raster file with a spatial resolution of 100 m × 100 m, and interpolated or resampled the above data into the raster file to obtain a continuous meteorological data surface, covering the study area.

#### 3.5.3. Grid Center Coordinate Transformation

Since traditional machine learning models did not consider the spatial autocorrelation of data, we constructed spatial dummy variables based on the method presented by Yang et al. [25]. In order to capture local effects, the longitude and latitude coordinates of each grid center point were converted to Cartesian coordinates, so that the parameters of the model were estimated at a local location rather than in the global space. This method constructs a local model for a specific small area, which can better capture the changes in the geographic spatial pattern with a high spatial resolution. The transformation formula is as follows:

(2) $$g_{i}=\left[\begin{array}{c} g_{xi}\\ g_{yi}\\ g_{zi} \end{array}\right]=\left[\begin{array}{c} R\sin\theta \cos\varphi \\ R\sin\varphi \\ R\cos\theta \cos\varphi \end{array}\right]$$

where φ is latitude, θ is longitude, and R is the radius of the earth. The purpose of geospatial coding is to measure the relative compactness of space. Therefore, in this study, R is set to 1 for normalization. In the predictor variables, the variables of $g_{x}$, $g_{y}$, and $g_{z}$ are abbreviated as x, y, and z.

#### 3.5.4. Calculating Land Use Raster Statistics Within the Buffer Zone and Matching Target Variables and Prediction Variables

For the sampling point data within the same day and hour, the corrected PM_2.5_ data of the sampling points in the same grid were averaged as the PM_2.5_ concentration of the grid for that day and hour. For each grid with PM_2.5_ concentration data within each hour, circular buffers with radii of 50 m, 250 m, 500 m, 750 m, 1000 m, 1250 m, 1500 m, 1750 m, and 2000 m were constructed, with the center of the grid as the center. The number of grids corresponding to the various land use types in each buffer was counted, and the numbers of different land use type grids in the buffers with different radii were used as the different prediction variables to match the PM_2.5_ values of the corresponding grid.

According to the hourly period corresponding to each grid, the grid values of the six types of meteorological data per hour within the period and covering the center of the grid were matched with the PM_2.5_ value of the grid where the center of the grid was located. This procedure allowed us to identify 170 locations and 31 h, with concentration estimates for model development, and a total of 1600 sample data points were ultimately obtained.

### 3.6. Stacking Model and Explainable Methods

#### 3.6.1. Modeling Approach

The stacking method is a powerful and complex technique in the field of ensemble learning within the machine learning technique. It is designed to enhance the predictive performance of machine learning models by leveraging the strengths of diverse base models and combining their predictions through a meta-model. The fundamental concept behind stacking lies in constructing a two-level learning process. At the first level, a set of diverse base models are trained on the input data, and each model employs different algorithms or feature representations. These base models generate individual predictions for the target variables. At the second level, the meta-model trains the prediction results of the base models. This way, the patterns and relationships among the outputs of each base model can be utilized to generate an improved overall prediction, thereby obtaining a final, more accurate prediction [26].

This study employs three different base model algorithms: random forest, XGBoost and CatBoost. These are used to train the three base models, respectively. The ridge regression model is used as the meta-model to construct a stacking model. Ridge regression is an improved linear regression algorithm that was specifically designed to handle multicollinearity (high correlation between features). It improves the generalization ability of the model and prevents overfitting by adding a penalty term that is proportional to the L2 norm of the model coefficients and to the loss function. Here, we provide a brief introduction to these three base model algorithms.

The random forest algorithm is a bagging algorithm in ensemble learning that uses decision trees as estimators. It combines multiple decision trees, where each dataset is generated by random sampling with replacement, and at each split, a random subset of features is selected as the input. In regression problems, the final result is obtained by averaging the predictions of multiple regression trees.

XGBoost (eXtreme Gradient Boosting) is an ensemble learning algorithm developed by Chen et al. [27]. It employs the gradient descent method to optimize the model by combining multiple weak learners (decision trees) into a strong learner. In this process, each subsequent learner learns the difference between the prediction of the previous base learner and the actual value. Through the learning of multiple learners, the difference between the model’s predictions and the actual values is continuously reduced, thereby improving prediction accuracy.

CatBoost is a GBDT framework that uses symmetric decision trees (oblivious trees) as base learners, featuring fewer parameters, native support for categorical variables, and high accuracy. It addresses the issues of gradient bias and prediction shift, thereby reducing overfitting and improving both the accuracy and generalization ability of the algorithm.

Hyperparameters need to be set before the machine learning model is trained. Hyperparameters are parameters that are used to control the behavior of the algorithm when building a model. These parameters cannot be learned from the normal training process. They need to be assigned before training the model. At present, there are four mainstream hyperparameter-tuning methods: traditional or manual parameter tuning, grid search, random search, and Bayesian search.

#### 3.6.2. Evaluation of Model Performance

The performance of the model is evaluated using K-fold cross-validation on the sample set. K-fold cross-validation is a model evaluation method that randomly divides the dataset into K roughly equal subsets. In each fold, K-1 subsets are used as training sets, and the remaining 1 subset is used as a test set. This process is repeated K times, each time using a different subset as the test set and the others as the training set. In each fold of validation, a new model is trained, and its evaluation indicators are calculated. The final value of each evaluation indicator of the K-fold cross-validation is the average of all K validations. This makes full use of the dataset, and the results are more reliable and helps avoid overfitting.

K-fold cross-validation R^2^, mean absolute percentage error (MAPE), root mean square error (RMSE), and mean absolute error (MAE) are selected as evaluation indicators.

(1) Coefficient of determination (R^2^):

(3) R2=1−∑i=1n(yi−yi^)∑i=1n(yi−yi_)

The coefficient of determination (R^2^) is an indicator of the goodness of fit of the regression model, indicating the degree to which the independent variable explains the variation of the dependent variable. The value of R^2^ is between 0 and 1; the larger the value, the better the model fits the data.

(2) Mean absolute percentage error (MAPE):

(4) $$MAPE=\frac{1}{n}\sum_{i=1}^{n}\left|\frac{y_{ture,i}-y_{pred,i}}{y_{ture,i}}\right|\times 100\%$$

The mean absolute percentage error measures the percentage of error between the predicted value and the true value, suitable for evaluating the accuracy of regression tasks.

(3) Root mean square error (RMSE):

(5) $$RMSE=\sqrt{\frac{1}{N}{\sum \left(y_{i}-\overset{\wedge }{y_{i}}\right)}^{2}}$$

where $y_{i}$ is the actual observation value, y^i is the model prediction value, N is the sample size, and RMSE squares the difference to ensure that all errors are positive numbers, while giving higher penalties to larger errors. The smaller the value, the higher the prediction accuracy of the model.

(4) Mean absolute error (MAE)

(6) MAE=1n∑i=1nyi−y^i

The mean absolute error is a commonly used evaluation indicator in regression models, which aims to measure the average difference between the model’s predicted value and the true value. Compared with other error metrics, the MAE does not consider the direction of the error (that is, it does not square the positive and negative errors), but simply calculates the average of the absolute values of the errors, which can particularly emphasize larger errors.

#### 3.6.3. Explainability Method Based on SHAP

Due to the black-box nature of machine learning models, we use the SHAP method to evaluate the feature importance of each feature in the model and increase the explainability of the model. Based on the feature importance results of the three base models, and after multiple rounds of feature combination training, we selected 81 features that did not lead to a decrease in accuracy. Since the XGBoost model achieved the best performance among the three base models, we conducted an explainability analysis based on the SHAP feature importance results of the XGBoost model. SHAP is a method for post-model explanation. Its core idea is to calculate the marginal contribution of features to the model output and then explain the “black-box model” from both the global and local levels. SHAP constructs an additive explanatory model in which all features are considered to be “contributors”. For each predicted sample, the model generates a predicted value, and the SHAP value is the value assigned to each feature in the sample. The basic idea is to calculate the marginal contribution of a feature when it is added to the model; then, one takes into account the different marginal contributions of the feature in all feature sequences, and take the average, which is the SHAP baseline value of the feature. The SHAP method can also be used to determine whether the relative high and low values of each feature have favorable or unfavorable effects on the target variable.

## 4. Results

### 4.1. Correction Results of Low-Cost Sensors

The variables and parameters of the multiple linear regression correction models for each low-cost sensor, as well as the R^2^, RMSE, and MRE of the models, are shown in Table 5.

The adjusted R^2^ values of these linear regression models were high and the RMSE values were all below 5 μg/m^3^ (which is within the low error range for estimating PM_2.5_ concentration [24]). The MRE values were slightly high but within an acceptable range. The possible reason was that most of the monitoring values of the PM_2.5_ concentration during the correction data collection period were low.

The descriptive statistics results of all models are shown in Table 6.

In these models, the coefficients and constants were statistically significant (*p* < 0.05) except for the constant term of one of the models. Since these models also had high adjusted R^2^, low RMSE, and acceptable MRE, we used these multiple linear regression models to correct the effective low-cost sensor data at each sampling point.

Statistics of the low-cost sensor monitoring data during co-location before and after correction, together with the monitoring data from the National Environmental Monitoring Station instruments, are presented in Table 7.

It can be seen that the monitoring quality of different low-cost sensors is different before correction, and the minimum, maximum, and average values are not equal. The maximum and average values are much higher than those of the national environmental monitoring station instrument. After correction, the difference in monitoring quality between different low-cost sensors is reduced, and they are all very close to the data of the national environmental monitoring station instrument. Combined with the low RMSE values and acceptable MRE values of each correction model, as seen in Table 5, it can be concluded that the correction effect is good.

### 4.2. Stacking Model Evaluation Results

#### 4.2.1. Evaluation Results of the Model Based on Corrected Low-Cost Sensor Data

**Figure 6:**
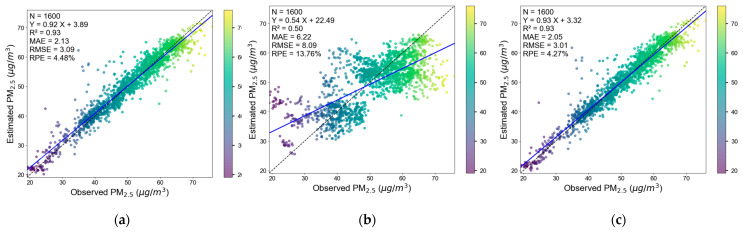
Evaluation scatter plots: 5-fold CV based on sampling points (**a**); 5-fold CV based on time (**b**); 5-fold CV based on space (**c**).

Figure 6 shows the scatter plots of the evaluation results of the three 5-fold cross-validation of the model based on the corrected low-cost sensor data.

For the 5-fold cross-validation based on sampling points, the training set and test set are divided according to different sampling points to evaluate the prediction ability of unsampled points. The results show that the model performance evaluated by this method is relatively high, with an RMSE of 3.09 and an R^2^ of 0.93, indicating that the model has a strong fitting ability on randomly distributed data. Another possible reason for such a high result is that the sampling between the sample points may be highly similar in space and time, resulting in the possibility that the samples in the training set and the test set are similar in space and time.

For the 5-fold cross-validation based on time, the training set and the test set are divided according to time to evaluate the prediction ability of unsampled hours. The evaluation indicators are significantly lower than those of the other two cross-validations, with RMSE increasing to 8.09, R^2^ decreasing to 0.50, and RPE and MAE also increasing significantly, indicating that the model has poor extrapolation performance in time. The possible reason is that we only sampled for 5 days, so the number of hours of data collected was relatively small, meaning that the sampling period may not have been long enough to capture enough of the hourly variation mechanism of PM_2.5_.

For the 5-fold cross-validation based on space, the training set and the test set are divided according to different sampling grid positions to evaluate the prediction ability of unsampled grid spatial positions. The RMSE is 3.01 and the R^2^ reaches 0.93, indicating that the model has good spatial migration ability and strong spatial generalization ability.

#### 4.2.2. Evaluation Results of the Model Based on Uncorrected Low-Cost Sensor Data

**Figure 7:**
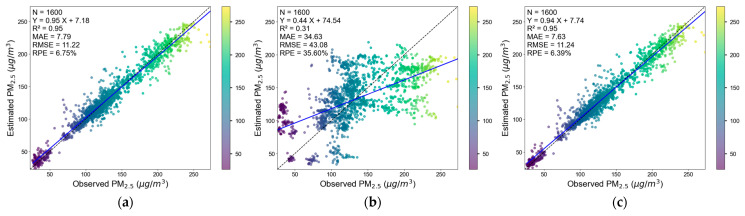
Evaluation scatter plots of the PM_2.5_ uncorrected model: 5-fold CV of sampling points (**a**); 5-fold CV based on time (**b**); 5-fold CV based on space (**c**).

Figure 7 shows the scatter plots of the evaluation results of the three 5-fold cross-validation of the model based on uncorrected low-cost sensor data.

Compared with the evaluation results of the model based on the corrected low-cost sensor data, the 5-fold cross-validation R^2^ (based on the sampling points) and the space of the model (based on the uncorrected data) are even slightly higher than those of the model what was based on the corrected low-cost sensor data. However, the RMSE values of the three cross-validations are much higher than that of the model that was based on the corrected low-cost sensor data. Moreover, the 5-fold cross-validation R^2^ based on time is significantly lower than that of the model that was based on the corrected low-cost sensor data.

From the above analysis results, it can be seen that, because low-cost sensors can provide sampling with higher spatial density, the accuracy of the spatial CV is not significantly lower than that of the sampling point-based CV. The prediction fit of the model based on corrected low-cost sensor data and that of the model based on uncorrected data from unseen sampling points and spaces is similar. However, the RMSE, MAE, etc., of the model based on the uncorrected data are higher; this is because the monitoring readings of the uncorrected low-cost sensors are significantly higher than those of the national environmental monitoring station instruments under the same environment, and the data fluctuations are greater.

#### 4.2.3. Independent Verification of Prediction Results and National Environmental Monitoring Station Data

By predicting the PM_2.5_ concentration at the Wuchang Ziyang National Environmental Monitoring Station, which is closest to the study area and is not involved in the correction (the Hankou Jiangtan National Environmental Monitoring Station is involved in the correction), the Pearson correlation coefficient between the prediction results of the model based on the corrected low-cost sensor data and the national environmental monitoring station data is 0.635 (*p* < 0.001), and the RMSE is 16.759 μg/m^3^. The Pearson correlation coefficient between the prediction results of the model based on the uncorrected low-cost sensor data and the national environmental monitoring station data is 0.625 (*p* < 0.001), and the RMSE is 72.068 μg/m^3^. It can be seen that the prediction results of the model have a high degree of credibility, and the corrected low-cost sensor data significantly reduce the prediction uncertainty.

#### 4.2.4. Feature Importance

**Figure 8:**
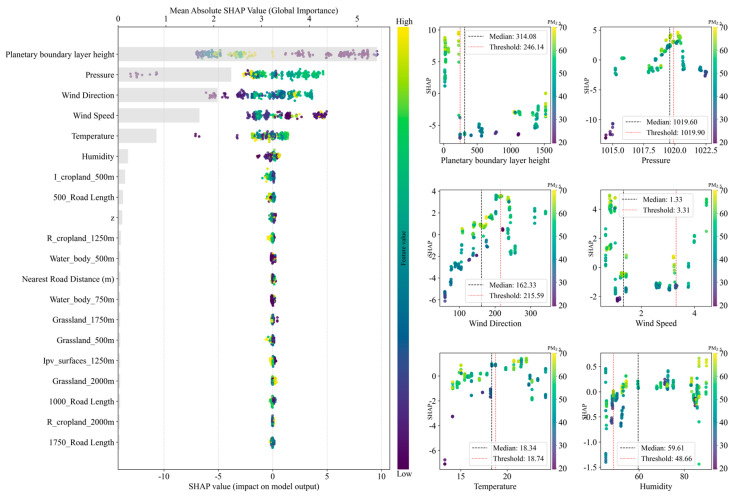
SHAP feature importance statistics plot (top 20 features). The left side of the plot is the SHAP summary plot, and the right side shows the SHAP dependency plots.

The feature importance of each feature based on the SHAP method is shown in Figure 8.

As can be seen from the SHAP summary plot, the most important feature is the planetary boundary layer height, followed by pressure, wind direction, wind speed, temperature, and humidity; these are all features with hourly time resolution, and the importance of the remaining features is relatively low. The remaining features are mostly land use types, including buffer road length, distance to the nearest road, and z coordinate in Cartesian coordinates; these are all static features with insufficient time resolution. This indicates that, for predictions with ultrahigh spatiotemporal resolution, high spatial resolution and simultaneous high temporal resolution are conducive to enhancing the contribution of a feature to a model; thus, such features are likely to affect the performance of the model.

A positive or negative SHAP value represents the direction of the feature’s influence on the target variable. A positive value represents a positive impact on the target variable, and a negative value represents a negative impact. A higher planetary boundary layer height basically has a negative impact on the PM_2.5_ concentration; that is, the higher the planetary boundary layer height, the lower the PM_2.5_ concentration will be, and the lower the planetary boundary layer height, the higher the PM_2.5_ concentration will be. Next are pressure and wind direction. The lower the pressure, the lower the PM_2.5_ concentration will be. For this study area, the smaller the wind direction angle, the lower the PM_2.5_ concentration will be. The larger the angle, the higher the PM_2.5_ concentration will be. The wind in the north direction is 0° and the wind direction increases in a clockwise direction; this finding, combined with the SHAP dependency plot, indicates that a westerly wind has an aggravating effect on the PM_2.5_ concentration in the study area; meanwhile, an easterly wind has a reducing effect on the PM_2.5_ concentration in the study area. The effects of the other features on the target variable are similar to those of the above principles.

### 4.3. Spatiotemporal Analysis

The hourly prediction data, daily prediction data, and all prediction data for 5 days from 31 October 2024, to 4 November 2024, were averaged, and visualization mapping and PM_2.5_ concentration statistics were conducted.

**Figure 9:**
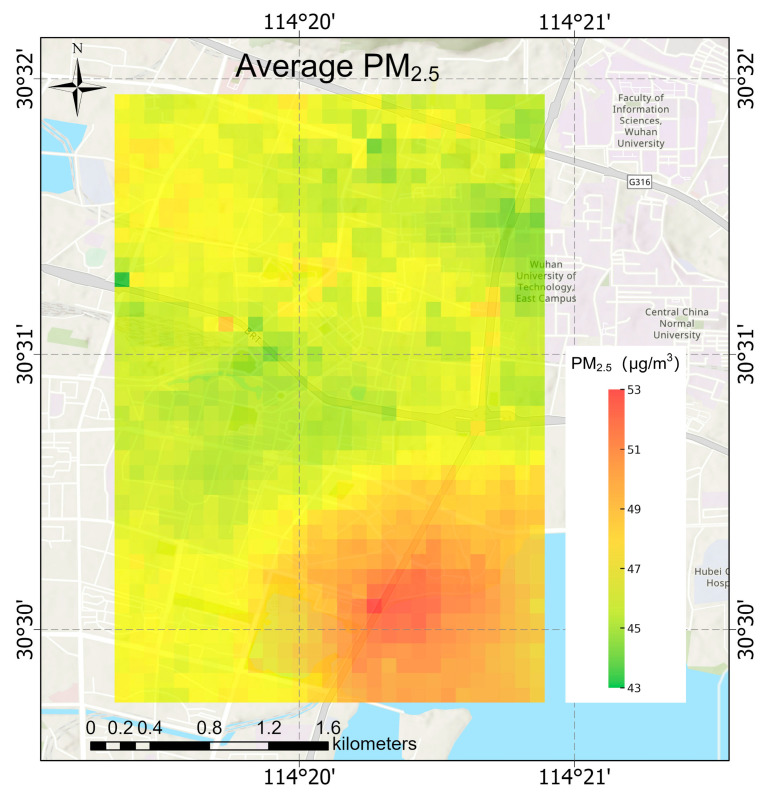
Average PM_2.5_ concentration distribution map from 31 October to 4 November.

The average concentration distribution map of PM_2.5_ from 31 October to 4 November is shown in Figure 9. The descriptive statistics of the average concentration of PM_2.5_ from 31 October to 4 November are shown in Table 8.

From Figure 9, it can be observed that PM_2.5_ concentrations are generally higher at major road intersections and congested traffic sections, reflecting the fact that vehicular traffic is an important contributor to PM_2.5_ levels. In areas with distinct elevated terrain, PM_2.5_ concentrations are relatively lower near the highest points. Land use types, water bodies, and dense greenbelts play clear buffering roles in reducing PM_2.5_ concentrations, particularly along water bodies with high greenbelts, like that located at the southwest corner of the area with the highest PM_2.5_ levels.

**Figure 10:**
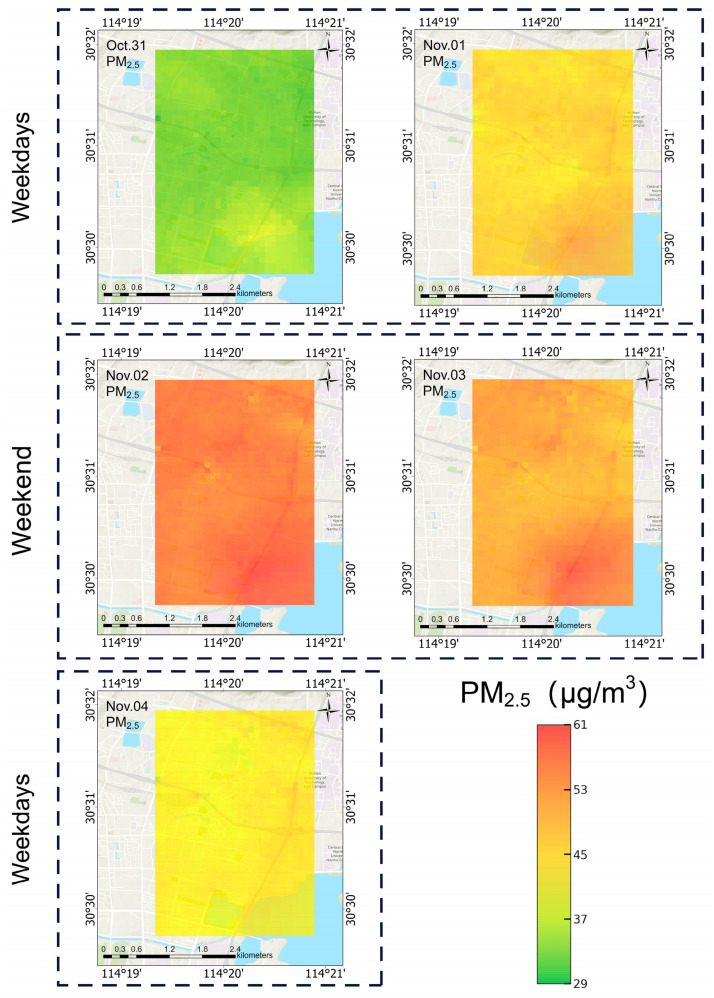
Daily PM_2.5_ concentration distribution map from 31 October to 4 November.

**Figure 11:**
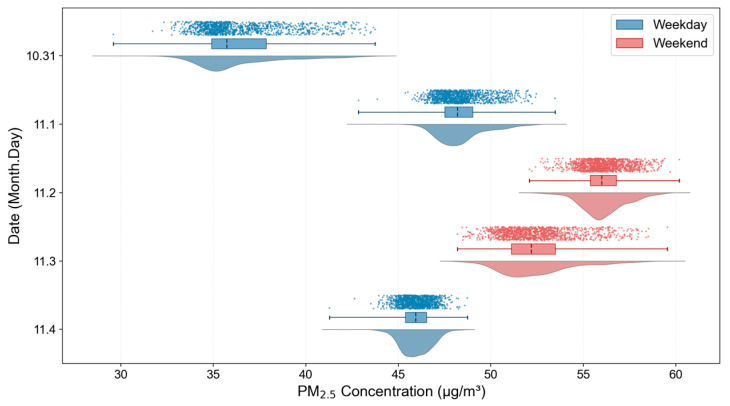
Daily PM_2.5_ concentration change statistics in 10.31–11.4 range.

The daily concentration distribution map of PM_2.5_ from 31 October to 4 November is shown in Figure 10. The statistical results of the daily change in PM_2.5_ concentration from 31 October to 4 November are shown in Figure 11.

31 October (Thursday) was a weekday, and the PM_2.5_ concentration in the study area was low, indicating that pollution was low. November 1 (Friday) was also a weekday, and the concentration increased, with emissions increasing in the later part of the working day. During the weekend of November 2 (Saturday) and 3 (Sunday), the concentration in the region increased significantly, indicating that there were more activities taking place, such as weekend travel, meaning that pollutant emissions increased significantly. 4 November (Monday) was a weekday, and the concentration fell, indicating that the adjustment of the activity pattern of the new week’s working day led to a decrease in emissions.

**Figure 12:**
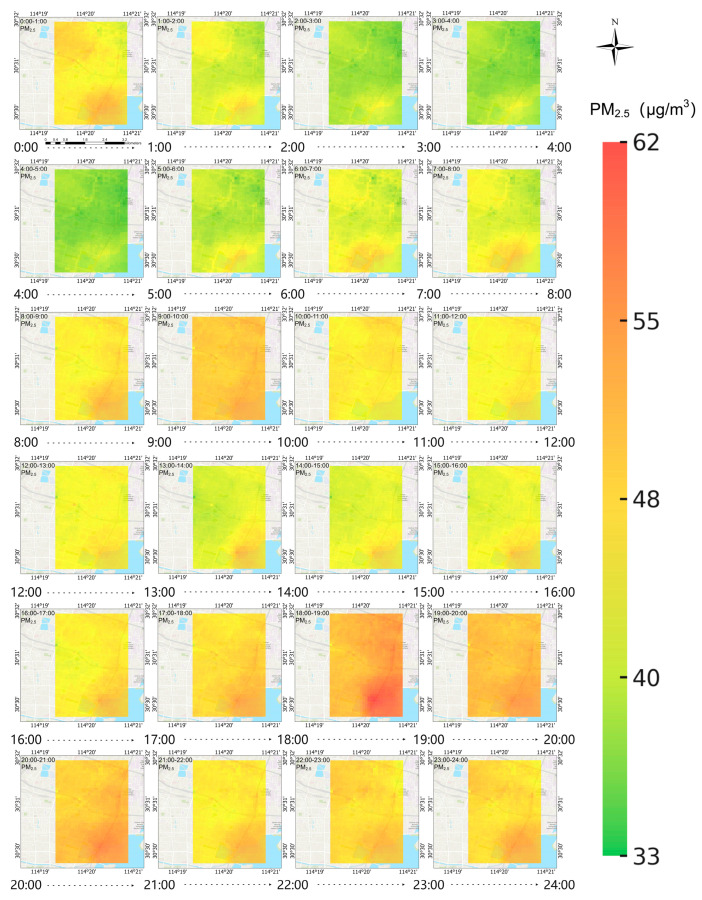
Average hourly PM_2.5_ concentration distribution map.

**Figure 13:**
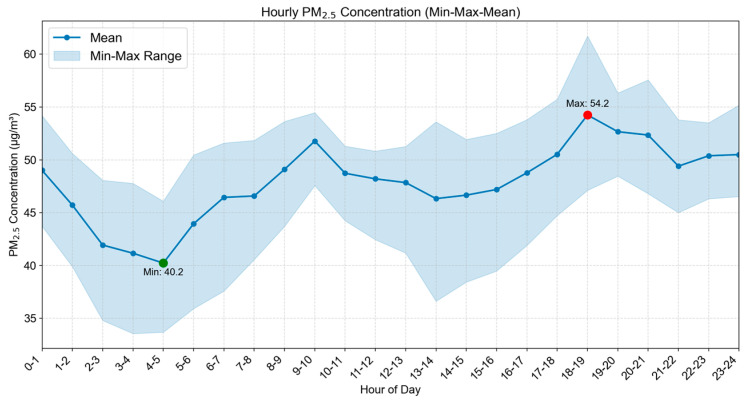
Average hourly PM_2.5_ concentration change statistics.

The average hourly concentration distribution of PM_2.5_ from 31 October to 4 November is shown in Figure 12. The statistical results of the average hourly change of PM_2.5_ concentration from 31 October to 4 November are shown in Figure 13.

From the 24-h PM_2.5_ concentration variation characteristics, from early morning to morning (2:00–5:00), human activities are at a low, and pollutant emissions are relatively low; in the morning (5:00–12:00), with the increase in morning rush hour traffic flow and the start of various activities, pollutant emissions increase and concentrations rise. PM_2.5_ pollution is most serious from 9:00 to 10:00, and most areas reach around 50 μg/m^3^; from noon to afternoon (12:00–16:00), the planetary boundary layer height increases, and the concentration shows an overall downward trend, especially from 14:00 to 16:00, where it remains at a relatively low level; PM_2.5_ pollution is relatively severe from the evening to the night (16:00 to 24:00), with 18:00 to 19:00 being the period of the day with the highest pollution. In many areas, pollution exceeds 55 μg/m^3^. The possible reasons for this are the superimposition of evening rush hour traffic emissions and various continuous activities. Moreover, the atmosphere is generally more stable at night, and the diffusion conditions deteriorate, causing pollutants to accumulate rapidly. Subsequently, pollutant concentrations gradually decline as human activities decrease, but they remain at a relatively high level. Overall, the changes in PM_2.5_ concentration in the study area show significant spatiotemporal coupling characteristics with the intensity of human activities and atmospheric diffusion conditions, and the PM_2.5_ concentration has an obviously high spatiotemporal heterogeneity in local areas and within a short period of time.

### 4.4. Spatial Autocorrelation Analysis

Spatial autocorrelation analyses can reveal the intrinsic structure and patterns of data in space. Through global and local indicators, we can gain insights into the similarities or differences between regions.

The global Moran’s I for PM_2.5_ concentration was significantly positive across all hourly and daily intervals during the study period. Hourly indices were generally above 0.83, mostly ranging between 0.89 and 0.96, with only a few time points were slightly below 0.85. All the daily indices also exceeded 0.78, peaking at 0.959 on 31 October, with an overall multi-day average of 0.932. All *p*-values were less than 0.001, indicating statistically significant results. The corresponding z-scores substantially exceeded the critical value of 2.58 (α = 0.01), further confirming the reliability of the spatial autocorrelation. The expected value of I, denoted as E[I], remained constant at −0.000842 across all periods, and the variance was also stable at approximately 0.000883, suggesting a robust model specification and a well-defined spatial weight matrix. These results demonstrate strong positive spatial autocorrelation and clustering of PM_2.5_ concentrations within the study area—that is, high values tend to cluster with other high values, and low values with low values, indicating a clear pattern of spatial homogeneity. This persistent high degree of clustering suggests that PM_2.5_ pollution exhibits not only local effects but also considerable regional spillover effects.

In addition, we conducted a local spatial autocorrelation analysis (LISA) by calculating the local Moran’s Index and visualizing the results of clustering and outlier analyses, which were used to detect local hot or cold spots. We conducted local spatial autocorrelation analyses on the total average, daily average, and hourly average PM_2.5_ concentrations during the study period, respectively.

**Figure 14:**
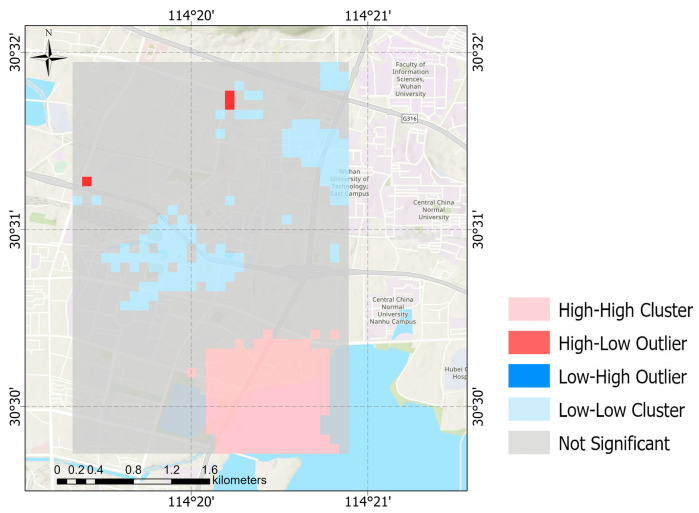
Local spatial autocorrelation map of total average concentration of PM_2.5_.

**Figure 15:**
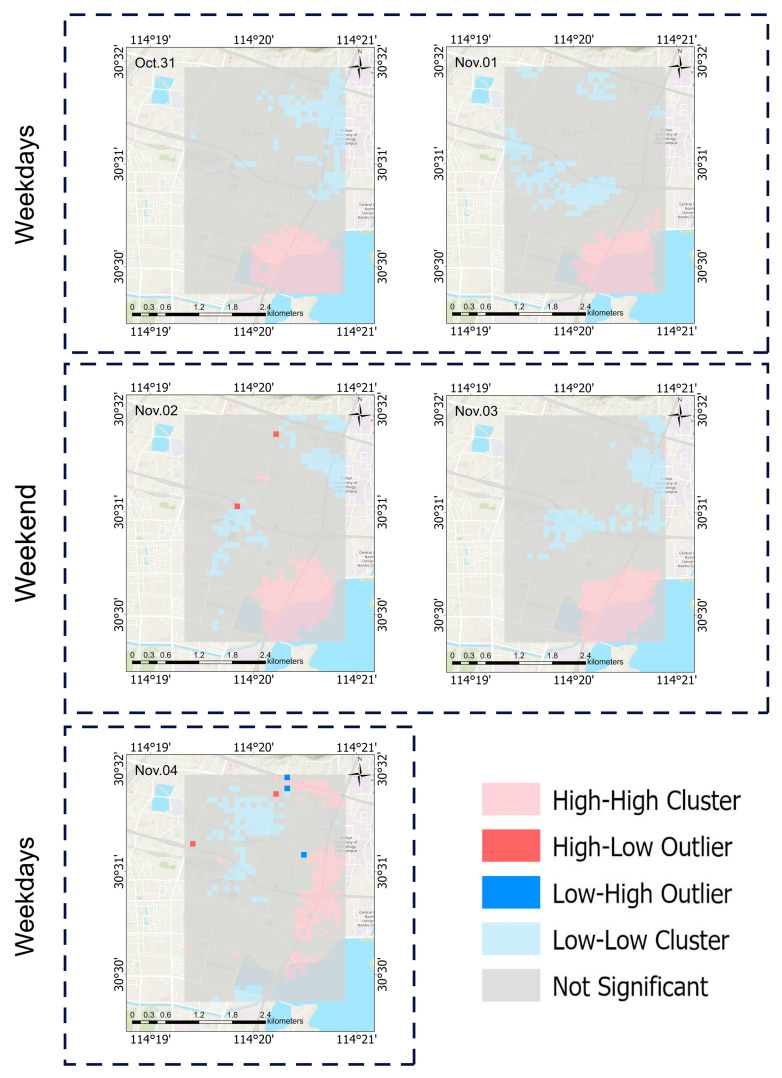
Local spatial autocorrelation map of the daily average concentration of PM_2.5_.

**Figure 16:**
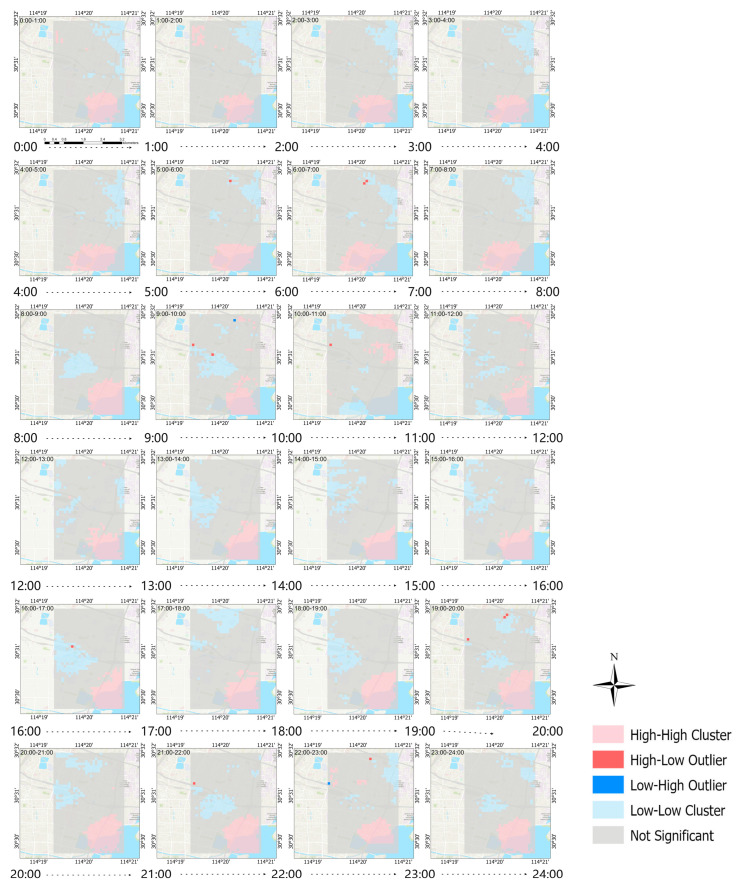
Local spatial autocorrelation map of multi-day hourly average concentration of PM_2.5_.

It can be seen from Figure 14, Figure 15 and Figure 16 that, except for during 10:00–11:00 on any given day and on the Monday during the 5-day study period, the southeastern part of the study area is a hot spot at all other times. This might be because the area has a large volume of traffic and is prone to slow passage, resulting in the continuous accumulation and diffusion of PM_2.5_ and thus affecting the surrounding areas. The fact that there were no hotspots in this area during several periods might be related to human passage habits. Cold spots were found to often occur to the north of the university in the study area and in the higher areas, with raised terrains. This might be because the road to the north of the university is a wide, major road, with relatively smooth traffic, making it less likely for PM_2.5_ to accumulate. In the higher areas with raised terrains, there is less traffic, and PM_2.5_ may fall to lower areas.

## 5. Discussion

### 5.1. Limitations

The number of sampling days and hours is too small, resulting in poor hourly cross-validation evaluation results, which to some extent affects the performance of the model for hourly prediction. Future work can increase the number of sampling days and the number of sampling hours per day.

The sampling area was relatively small, covering about 10 square kilometers. The role of an important predictor variable that can capture vertical and regional variability—AOD satellite remote sensing data with a 1 km × 1 km spatial resolution—was therefore limited in the modeling process. This was one of the reasons why we did not incorporate AOD satellite data into the model. Although dedicated correction was performed for the low-cost sensors, due to the limited size of the study area, the nearest national environmental monitoring station used for external validation was about 4 km away from the study area, which, to some extent, affected the validation of the prediction results. Future work could expand the study area to include, and preferably cover, multiple national environmental monitoring stations.

### 5.2. Future Works

In the future, more predictor variables with high spatiotemporal resolution could be included: high-spatiotemporal-resolution prediction variables also include satellite remote sensing images. Satellite remote sensing images have advantages: wide coverage, resampling potential, and fast update speed. They are also suitable for inclusion in high-spatiotemporal-resolution modeling datasets. For PM_2.5_ prediction, AOD (aerosol optical depth) data can be included. At present, there is a method for obtaining certain ultrahigh-spatial-resolution predictor variables; this involves utilizing densely distributed street-view images and applying deep-learning-based semantic segmentation approach for extracting different features from the images, thereby deriving street-level predictor variables [28,29]. Moreover, more advanced machine learning algorithms or deep learning algorithms can be applied to achieve more accurate predictions of air quality.

## 6. Conclusions

This study constructed a stacking model by integrating ultrahigh-density sampling data from low-cost sensors with multi-source predictor variables, achieving PM_2.5_ concentration prediction with a spatial resolution of 100 m × 100 m and an hourly temporal resolution within the city. After correction by multiple linear regression, the data quality of low-cost sensors has significantly improved and the monitoring quality difference has decreased, meaning that these data are more similar to the data from the national environmental monitoring station instruments. The model performs well in spatial generalization and random sample fitting. The R^2^ values based on space cross-validation and sampling points cross-validation are both 0.93. However, due to the short sampling duration of 5 days, the time extrapolation performance is weak, and the cross-validation R^2^ based on time is 0.50. The analysis of feature importance shows that high-frequency meteorological factors such as the planetary boundary layer height and atmospheric pressure make significant contributions to prediction, while static features such as land use type have relatively low importance, reflecting the key role of high-temporal-resolution features in ultrahigh-spatiotemporal-resolution prediction. The results of the spatiotemporal analysis show that the overall PM_2.5_ concentration on weekends is significantly higher than that during the week. The PM_2.5_ concentration has obvious local variability and varies according to the hours within a day. The PM_2.5_ concentration gradually increases with the arrival of the morning rush hour and gradually decreases in the afternoon, until it reaches the lowest value of the day. The evening rush hour is from 18:00 to 19:00, and during this time, the PM_2.5_ concentration reaches its maximum in the day and remains at a relatively high level until 1:00 a.m.

However, this study has some limitations, such as a short sampling period, a small study area, and a lack of national environmental monitoring stations within the study area. Future research could increase the sampling period and expand the study area. Furthermore, it could incorporate AOD satellite remote sensing data or high-resolution variables such as features extracted from street-level imagery. Finally, it could optimize low-cost sensor monitoring designs and employ more advanced algorithms to enhance model performance. Accordingly, it would be possible to provide better technical support for urban air pollution prevention and control and health risk assessments.

## Figures and Tables

**Table 1:** Monitoring schedule.

Monitoring Time	Date				
	10/31/2024	11/1/2024	11/2/2024	11/3/2024	11/4/2024
6am–7am	√	√	√		√
7am–8am	√	√	√		√
8am–9am	√	√	√		√
9am–10am		√			
11am–12am			√		√
12am–1pm			√		√
1pm–2pm			√		√
2pm–3pm			√		
4pm–5pm			√		√
5pm–6pm			√		√
6pm–7pm			√		√
7pm–8pm			√		
9pm–10pm			√		√
10pm–11pm			√		√

**Table 2:** Low-cost sensor correction hourly data statistics table.

Equipment Number	Effective Monitoring Hours (h)	Co-Location Start Time	Co-Location End Time
1	141	9/10/2024	10/30/2024
2	134	9/10/2024	10/30/2024
3	144	9/10/2024	10/30/2024
4	167	9/10/2024	10/30/2024

**Table 3:** Land cover data description.

Type Number	Variable Name
0	Filled value
10	Rainfed cropland
11	Herbaceous cover cropland
20	Irrigated cropland
51	Open evergreen broadleaved forest
52	Closed evergreen broadleaved forest
61	Open deciduous broadleaved forest (0.15 < fc < 0.4)
62	Closed deciduous broadleaved forest (fc > 0.4)
71	Open evergreen needle-leaved forest (0.15 < fc < 0.4)
72	Closed evergreen needle-leaved forest (fc > 0.4)
121	Evergreen shrubland
130	Grassland
181	Swamp
182	Marsh
183	Flooded flat
190	Impervious surfaces
200	Bare areas
210	Water body

**Table 4:** Predictor statistics.

Variable Name	Type of Data and Source	Predictor Variable Description	Unit	Buffer Zone Radius (m)
Filled value_radius m	Land use data (GLC_FCS30 fine land cover data, 2022)	Filled value	1	50, 250, 500, 750, 1000, 1250, 1500, 1750, 2000
R_cropland_radius m		Rainfed cropland	1	50, 250, 500, 750, 1000, 1250, 1500, 1750, 2000
Hc_cropland_radius m		Herbaceous cover cropland	1	50, 250, 500, 750, 1000, 1250, 1500, 1750, 2000
I_cropland_radius m		Irrigated cropland	1	50, 250, 500, 750, 1000, 1250, 1500, 1750, 2000
Oeb_forest_radius m		Open evergreen broadleaved forest	1	50, 250, 500, 750, 1000, 1250, 1500, 1750, 2000
Ceb_forest_radius m		Closed evergreen broadleaved forest	1	50, 250, 500, 750, 1000, 1250, 1500, 1750, 2000
Odb_forest_radius m		Open deciduous broadleaved forest	1	50, 250, 500, 750, 1000, 1250, 1500, 1750, 2000
Cdb_forest_radius m		Closed deciduous broadleaved forest	1	50, 250, 500, 750, 1000, 1250, 1500, 1750, 2000
Oen_forest_radius m		Open evergreen needle-leaved forest	1	50, 250, 500, 750, 1000, 1250, 1500, 1750, 2000
Cen_forest_radius m		Closed evergreen needle-leaved forest	1	50, 250, 500, 750, 1000, 1250, 1500, 1750, 2000
E_shrubland_radius m		Evergreen shrubland	1	50, 250, 500, 750, 1000, 1250, 1500, 1750, 2000
Grassland_radius m		Grassland	1	50, 250, 500, 750, 1000, 1250, 1500, 1750, 2000
Swamp_radius m		Swamp	1	50, 250, 500, 750, 1000, 1250, 1500, 1750, 2000
Marsh_radius m		Marsh	1	50, 250, 500, 750, 1000, 1250, 1500, 1750, 2000
Fld_flat_radius m		Flooded flat	1	50, 250, 500, 750, 1000, 1250, 1500, 1750, 2000
Ipv_surfaces_radius m		Impervious surfaces	1	50, 250, 500, 750, 1000, 1250, 1500, 1750, 2000
Bare areas_radius m		Bare areas	1	50, 250, 500, 750, 1000, 1250, 1500, 1750, 2000
Water body_radius m		Water body	1	50, 250, 500, 750, 1000, 1250, 1500, 1750, 2000
x	Geospatial variables (Cartesian coordinates converted from latitude and longitude)	g_x_	1	NA
y		g_y_	1	NA
z		g_z_	1	NA
Radius_Road Length	Road network data (Open Street Map, 2024)	The length of all major roads within the buffer zone of the specified radius	m	50, 250, 500, 750, 1000, 1250, 1500, 1750, 2000
Nearest Road Distance (m)		The minimum distance from the center of the raster corresponding to the buffer zone to the nearest major road	m	50, 250, 500, 750, 1000, 1250, 1500, 1750, 2000
Temperature	Meteorological station data (Environmental meteorological data service platform, China ground meteorological station hourly data standard version)	The temperature monitored by meteorological stations	℃	NA
Pressure		The air pressure monitored by meteorological stations	Pa	NA
Wind Speed		2-min average wind speed	m·s^−1^	NA
Wind Direction		2-min average wind direction	degree	NA
Humidity		The relative humidity monitored by meteorological stations	%	NA
Planetary boundary layer height	Reanalyzing meteorological data (ERA5 reanalysis dataset, ERA: European Centre for Medium-Range Weather Forecast Reanalysis)	Planetary boundary layer height	kilometer	NA

**Table 5:** Variables and parameters of the multiple linear regression correction models for each low-cost sensor, as well as the R^2^, RMSE, and MRE of the models.

Low-Cost Sensors	Equipment Number	Correction Equation	Adjusted R^2^	RMSE	MRE
SDL307	1	Corrected PM_2.5_ = PM_2.5_ × 0.265 + temperature × 0.268 − relative humidity × 0.1 + 6.378	0.927	4.129	10.98%
	2	Corrected PM_2.5_ = PM_2.5_ × 0.315 + temperature × 0.173 − relative humidity × 0.06 + 5.487	0.922	4.377	12.31%
	3	Corrected PM_2.5_ = PM_2.5_ × 0.229 − relative humidity × 0.159 + 17.48	0.926	4.728	10.69%
	4	Corrected PM_2.5_ = PM_2.5_ × 0.458 − temperature × 0.156 − relative humidity × 0.12 + 15.366	0.908	4.904	6.85%

**Table 6:** Model descriptive statistics results.

Low-Cost Sensors	Equipment Number	PM_2.5_		Temperature		Relative Humidity		Constant	
		Significance	VIF	Significance	VIF	Significance	VIF	Significance	VIF
SDL307	1	<0.001	1.153	<0.001	1.429	<0.001	1.267	0.043	<0.001
	2	<0.001	1.104	0.018	1.367	0.046	1.254	0.101	<0.001
	3	<0.001	1.057	-	-	<0.001	1.057	<0.001	<0.001
	4	<0.001	1.065	0.04	1.338	<0.001	1.345	<0.001	<0.001

**Table 7:** Statistics of low-cost sensor monitoring data during co-location before and after correction and the National Environmental Monitoring Station instruments’ monitoring data.

Low-Cost Sensors	Equipment Number	Min (μg/m^3^)	Max (μg/m^3^)	Mean (μg/m^3^)
SDL307 after correction	1	5.94	272.72	58.06
	2	4.86	220.08	51.46
	3	5.26	282.35	81.20
	4	2.84	139.32	46.39
SDL307 before correction	1	5.72	73.08	21.41
	2	5.93	71.61	21.66
	3	6.04	66.99	25.03
	4	4.42	65.02	24.40
National Environmental Station Instruments	-	1.00	77.00	23.21

**Table 8:** Descriptive statistics of average PM_2.5_ concentration from 31 October to 4 November.

	Min	Max	Mean
PM_2.5_ Concentration (μg/m^3^)	43.1004	52.7418	47.9068

## Data Availability

The raw data supporting the conclusions of this article will be made available by the authors on request.

## References

[1] Enkhjargal O., Lamchin M., Chambers J., You X.Y. (2023). Linear and Nonlinear Land Use Regression Approach for Modelling PM_2.5_ Concentration in Ulaanbaatar, Mongolia during Peak Hours. Remote Sens..

[2] Ma Z., Hu X., Huang L., Bi J., Liu Y. (2014). Estimating Ground-Level PM_2.5_ in China Using Satellite Remote Sensing. Environ. Sci. Technol..

[3] Brokamp C., Jandarov R., Hossain M., Ryan P. (2018). Predicting Daily Urban Fine Particulate Matter Concentrations Using a Random Forest Model. Environ. Sci. Technol..

[4] Zou B., Chen J., Zhai L., Fang X., Zheng Z. (2017). Satellite Based Mapping of Ground PM_2.5_ Concentration Using Generalized Additive Modeling. Remote Sens..

[5] Feng S., Gao D., Liao F., Zhou F., Wang X. (2016). The Health Effects of Ambient PM_2.5_ and Potential Mechanisms. Ecotoxicol. Environ. Saf..

[6] Wu J., Xie W., Li J. (2016). Application of Land Use Regression Model in the Study of Spatiotemporal Variation of Atmospheric Pollution. Environ. Sci..

[7] Cheng L.X., Tao J.H., Yu C., Zhang Y., Fan M., Wang Y.P., Chen Y.L., Zhu L.L., Gu J.B., Chen L.F. (2021). Research on Remote Sensing Retrieval of Tropospheric NO_2_ Column Concentration Using GF-5 Satellite Atmospheric Trace Gas Differential Absorption Spectrometer. J. Remote Sens..

[8] Chao C.-Y., Zhang H., Hammer M., Zhan Y., Kenney D., Martin R.V., Biswas P. (2021). Integrating Fixed Monitoring Systems with Low-Cost Sensors to Create High-Resolution Air Quality Maps for the Northern China Plain Region. ACS Earth Space Chem..

[9] Lee H.J. (2019). Benefits of High Resolution PM_2.5_ Prediction using Satellite MAIAC AOD and Land Use Regression for Exposure Assessment: California Examples. Environ. Sci. Technol..

[10] Messier K.P., Chambliss S.E., Gani S., Alvarez R., Brauer M., Choi J.J., Hamburg S.P., Kerckhoffs J., LaFranchi B., Lunden M.M. (2018). Mapping Air Pollution with Google Street View Cars: Efficient Approaches with Mobile Monitoring and Land Use Regression. Environ. Sci. Technol..

[11] Wu P., Song Y. (2022). Land Use Quantile Regression Modeling of Fine Particulate Matter in Australia. Remote Sens..

[12] Minet L., Liu R., Valois M.-F., Xu J., Weichenthal S., Hatzopoulou M. (2018). Development and Comparison of Air Pollution Exposure Surfaces Derived from On-Road Mobile Monitoring and Short-Term Stationary Sidewalk Measurements. Environ. Sci. Technol..

[13] Geng G., Murray N.L., Chang H.H., Liu Y. (2018). The Sensitivity of Satellite-Based PM_2.5_ Estimates to Its Inputs: Implications to Model Development in Data-Poor Regions. Environ. Int..

[14] Zeng X., Ruan F., Peng Y. (2019). Spatial Distribution of Health Effects of PM_2.5_ Pollution in China Based on Spatial Grid Scale. China Environ. Sci..

[15] Jain S., Presto A.A., Zimmerman N. (2021). Spatial Modeling of Daily PM_2.5_, NO2, and CO Concentrations Measured by a Low-Cost Sensor Network: Comparison of Linear, Machine Learning, and Hybrid Land Use Models. Environ. Sci. Technol..

[16] Bi J., Burnham D., Zuidema C., Schumacher C., Gassett A.J., Szpiro A.A., Kaufman J.D., Sheppard L. (2024). Evaluating Low-Cost Monitoring Designs for PM_2.5_ Exposure Assessment with a Spatiotemporal Modeling Approach. Environ. Pollut..

[17] Huang Y. (2022). Research on Spatiotemporal Characteristics and Functional Data Analysis of PM_2.5_: Based on Data from 9 Monitoring Stations in Nanchang City. Master’s Thesis.

[18] Zhang S., Chen P., Zhang Y., Zhu C., Zhang C., Lu J., Wu M., Yang X. (2025). Estimating Hourly Surface PM_2.5_ Concentrations with Full Spatiotemporal Coverage in China Using Himawari-8/9 AOD and a Two-Stage Model. Atmos. Pollut. Res..

[19] Levy R.C., Mattoo S., Munchak L.A., Remer L.A., Sayer A.M., Patadia F., Hsu N.C. (2013). The Collection 6 MODIS Aerosol Products over Land and Ocean. Atmos. Meas. Tech..

[20] Chen T.Q., Guestrin C. XGBoost: A Scalable Tree Boosting System. Proceedings of the 22nd ACM SIGKDD International Conference on Knowledge Discovery and Data Mining (KDD ‘16).

[21] Shikhovtsev A.Y., Kovadlo P.G., Kiselev A.V., Eselevich M.V., Lukin V.P. (2023). Application of Neural Networks to Estimation and Prediction of Seeing at the Large Solar Telescope Site. Publ. Astron. Soc. Pac..

[22] Guo Y., Wu X., Qing C., Su C., Yang Q., Wang Z. (2022). Blind Restoration of Images Distorted by Atmospheric Turbulence Based on Deep Transfer Learning. Photonics.

[23] Yang Q., Kim J., Cho Y., Lee W.-J., Lee D.-W., Yuan Q., Wang F., Zhou C., Zhang X., Xiao X. (2023). A Synchronized Estimation of Hourly Surface Concentrations of Six Criteria Air Pollutants with GEMS Data. npj Clim. Atmos. Sci..

[24] Xu S., Zou B., Xiong Y., Wan N., Feng H., Hu C., Lin Y. (2021). High spatiotemporal resolution mapping of PM_2.5_ concentrations under a pollution scene assumption. J. Clean. Prod..

[25] Yang N., Shi H., Tang H., Yang X. (2022). Geographical and Temporal Encoding for Improving the Estimation of PM_2.5_ Concentrations in China Using End-to-End Gradient Boosting. Remote Sens. Environ..

[26] Benaida M., Abnane I., Idri A. (2026). Stacked ensembles for one-step and multi-step ahead BGL forecasting. Biomed. Signal Process. Control.

[27] Chen M., Xin J., Tang Q., Hu T., Zhou Y., Zhou J. (2024). Explainable machine learning model for load-deformation correlation in long-span suspension bridges using XGBoost-SHAP. Dev. Built Environ..

[28] Qi M., Hankey S. (2021). Using Street View Imagery to Predict Street-Level Particulate Air Pollution. Environ. Sci. Technol..

[29] Qi M., Dixit K., Marshall J.D., Zhang W., Hankey S. (2022). National Land Use Regression Model for NO2 Using Street View Imagery and Satellite Observations. Environ. Sci. Technol..

